# Inhibition of autophagic flux by S-nitrosylation of SQSTM1/p62 promotes neuronal secretion and cell-to-cell transmission of SNCA/α-synuclein in Parkinson disease and Lewy body dementia

**DOI:** 10.1080/27694127.2022.2076770

**Published:** 2022-05-30

**Authors:** Chang-ki Oh, Tomohiro Nakamura, Stuart A. Lipton

**Affiliations:** a Neurodegeneration New Medicines Center and Department of Molecular Medicine, The Scripps Research Institute, La Jolla, California; b Department of Neurosciences, University of California, San Diego, School of Medicine, La Jolla, California

**Keywords:** α-synuclein, autophagy, Lewy body dementia, p62, Parkinson’s disease, S-nitrosylation

## Abstract

Autophagy (in the form of macroautophagy) is the major intracellular protein quality control system for removal of damaged organelles and abnormally aggregated proteins. We and others have shown that dysregulated autophagic pathways contribute to accumulation and spread of misfolded proteins in many neurodegenerative disorders, including Parkinson disease (PD) and Lewy body dementia (LBD). Additionally, generation of excessive reactive oxygen and nitrogen species, such as nitric oxide (NO), accelerates neuronal and synaptic damage mediated, at least in part, via aberrant protein S-nitrosylation. Using cell-based models, including human induced pluripotent stem cell (hiPSC)-derived neurons, CRISPR-Cas9 technology, and transgenic PD/LBD mice, plus vetting in human postmortem brains, we found that S-nitrosylation of the autophagic receptor protein SQSTM1/p62 (forming SNO-SQSTM1/p62) inhibits autophagic flux, thus contributing to accumulation of misfolded SNCA/α-synuclein. Consequently, this impairment in autophagy increases extracellular vesicle-dependent secretion and spread of aggregated SNCA. Taken together, our evidence suggests that aberrant formation of SNO-SQSTM1/p62 represents a pathogenic event contributing not only to inhibition of autophagic flux and potentiation of neuronal damage, but also to propagation of α-synucleinopathy between cells in the diseased brain.

Macroautophagy (referred to here simply as autophagy) is a lysosomal degradative process in which damaged organelles and misfolded, aggregated proteins are eliminated to maintain cellular homeostasis. Among the autophagy regulating proteins, SQSTM1/p62 was the first mammalian protein identified as an autophagy receptor molecule, binding to both LC3 and ubiquitinated proteins through its LC3-interacting region (LIR) motif and ubiquitin-associated domain, respectively. Accordingly, SQSTM1/p62 acts as an autophagic cargo linker that delivers ubiquitinated misfolded proteins and organelles to phagophores during the autophagy-lysosomal degradation process. In contrast, an impairment in autophagy allows accumulation of potentially toxic cellular products, contributing to the pathogenesis of neurodegenerative disorders such as Parkinson disease (PD), Lewy body dementia (LBD), Alzheimer disease, Huntington disease, and amyotrophic lateral sclerosis. Accumulation of reactive oxygen species (ROS) and reactive nitrogen species (RNS), such as nitric oxide (NO), represents another contributing factor to neurodegenerative diseases. Along these lines, our group and others have demonstrated that aberrant protein S-nitrosylation, involving covalent reaction of NO-related species with a critical cysteine thiol (or thiolate anion) on target proteins, mediates in part the neurotoxic effects of RNS. In the study reviewed here [1], we demonstrated that S-nitrosylation of SQSTM1/p62 inhibits autophagic flux, promoting the spread and propagation of synucleinopathy under pathophysiologically-relevant conditions.

Initially, we found that S-nitrosylation of SQSTM1/p62 (forming SNO-SQSTM1/p62) is significantly elevated in SH-SY5Y cells exposed to the physiological NO donor, S-nitrosocysteine (SNOC) or to a PD-related toxin, rotenone, which triggers neurotoxic production of RNS/NO. Additionally, we observe an aberrant increase in the formation of SNO-SQSTM1/p62 *in vivo* in an SNCA/α-synuclein-overexpressing mouse model of PD/LBD. Moreover, we find elevated levels of SNO-SQSTM1/p62 in human iPSC-derived A9-type dopaminergic neurons (designated hiPSC-DA neurons) bearing an A53T mutation in the *SNCA* gene, which causes a familial form of PD, when compared to isogenic, gene-corrected (wild-type [WT]) control hiPSC-DA neurons. Notably, exposure of isogenic control hiPSC-DA neurons to rotenone also increases SNO-SQSTM1/p62 to levels comparable to those observed in untreated A53T hiPSC-DA neurons. Mechanistically, we found the predominant site of S-nitrosylation on SQSTM1/p62 occurs at cysteine 331, located in the LIR motif, resulting in increased binding of SQSTM1/p62 to LC3. A non-nitrosylatable SQSTM1/p62 mutant in which alanine is substituted for cysteine 331 (SQSTM1^C331A^) binds to LC3 in cells at comparable levels to SNO-SQSTM1/p62 formed after exposure to SNOC. Thus, non-nitrosylatable mutant SQSTM1/p62 phenocopies the effect of SNO-SQSTM1/p62 in this regard. To further characterize the enhanced ability of SNO-SQSTM1/p62 and SQSTM1^C331A^ binding to LC3, we generated an atomic structure of the LC3-SQSTM1/p62 complex using homology modeling. The model structure predicted that SNO formation or Ala substitution for SQSTM1/p62 Cys331 alters its local protein environment, increasing the electrostatic interaction between SQSTM1/p62 and LC3. Therefore, S-nitrosylation of SQSTM1/p62 at Cys331 (or substitution of Cys331 with Ala) would be expected to increase the binding of SQSTM1/p62 to LC3, as we observed.

Next, because non-nitrosylatable mutant SQSTM1/p62 mimics the effects of SNO-SQSTM1/p62 on the SQSTM1/p62-LC3 interaction, we generated an SH-SY5Y cell line with a C331A knockin mutation in the endogenous *SQSTM1* gene using CRISPR-Cas9-mediated homology-direct repair (HDR) methodology. Note therefore that usage of non-nitrosylatable mutant SQSTM1/p62 allowed us to investigate the possible pathophysiological role of enhanced SQSTM1/p62-LC3 interactions on autophagy without increasing NO production, which otherwise would have affected autophagy processes by additional mechanisms to SNO-SQSTM1/p62 formation. Using these knockin cells compared to isogenic controls, we found that autophagic flux is drastically attenuated in the SQSTM1^C331A^ mutants because of inhibition of fusion between autophagosomes and lysosomes, resulting in accumulation of autophagosomes.

Recent evidence suggests that inhibition of autophagic flux may lead to the secretion of abnormally folded SNCA in extracellular vesicles (EVs), contributing to cell-to-cell transmission of aberrant SNCA. We found that EVs released from SQSTM1^C331A^ SH-SY5Y cells contain increased levels of SNCA, SQSTM1/p62, and LC3-II compared to isogenic control cells. Additionally, we find significantly increased SNCA, SQSTM1/p62, and LC3-II in EVs released from A53T *SNCA* hiPSC-DA neurons, which also manifest high levels of SNO-SQSTM1/p62, compared to isogenic control hiPSC-DA neurons. Notably, the NOS (nitric oxide synthase) inhibitor l-nitro-arginine (l-NAME) decreases EV-mediated secretion of SNCA, consistent with the notion that NO-related species contribute to pathological release of aggregated SNCA via EVs.

Next, to validate if SNCA secreted in EVs is efficiently transported into other cells, we monitored uptake of extracellular SNCA using a dual-cell bimolecular fluorescence complementation platform. This assay utilizes two forms of SNCA: 1) SNCA linked to the N-terminal fragment of Venus, secreted from SQSTM1^C331A^ SH-SY5Y cells or isogenic WT control SH-SY5Y cells; and 2) SNCA conjugated to the C-terminal fragment of Venus, expressed in the recipient cells. Aggregation of these two forms of SNCA in the recipient cells then causes dimerization of the two Venus fragments, resulting in fluorescence. Using this approach, we find significantly increased cell-to-cell transmission of SNCA secreted from SQSTM1^C331A^ SH-SY5Y cells compared to WT cells, suggesting that the SQSTM1^C331A^ mutant, which phenocopies the inhibitory effects of SNO-SQSTM1/p62 on autophagic flux, increases SNCA secretion and promotes cell-to-cell transmission of SNCA.

Concerning the pathophysiological importance of the spread of SNCA, cognitive dysfunction in the context of LBD is thought to be associated with cell-to-cell transmission of misfolded, mutant SNCA. Because we found that SNO-SQSTM1/p62 facilitates this spread, we examined if enhanced levels of SNO-SQSTM1/p62 are present in human LBD brains. Indeed, we detect significantly increased (~2-fold) SNO-SQSTM1/p62 levels in postmortem cerebrocortical brain tissues obtained from human LBD patients compared to controls. This relative increase in SNO-SQSTM1/p62 in human LBD brain is similar to that found in our cell-based models and SNCA transgenic mice, consistent with the notion that pathophysiologically-relevant amounts of SNO-SQSTM1/p62 are present in human LBD brains.

Collectively, our study demonstrates that S-nitrosylation of SQSTM1/p62 inhibits autophagic flux, leading to an increase in SNCA secretion and cell-to-cell transmission, predominantly via EVs (**Figure 1**). Moreover, because SNO-SQSTM1/p62 is present in human LBD brain at similar levels as in our model systems, this same phenomenology probably occurs in LBD brain. Thus, SNO-SQSTM1/p62 may represent a novel therapeutic target for α-synucleinopathies and related neurodegenerative diseases.

**Figure 1.**
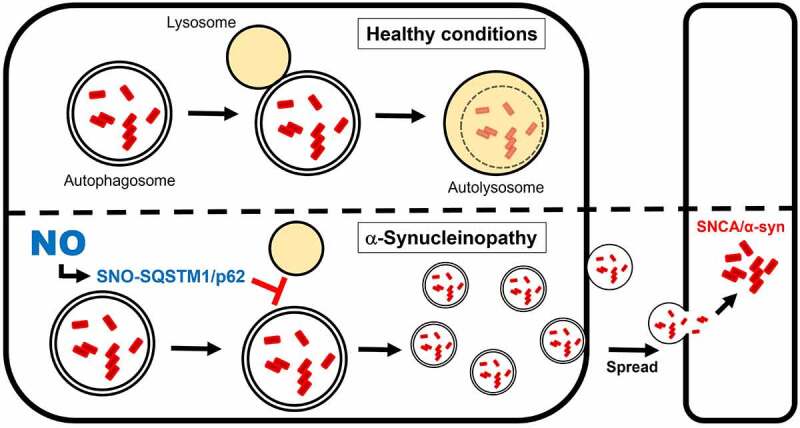
Schematic mechanism of SNO-SQSTM1/p62 (S-nitrosylated SQSTM1/p62)-dependent spread of aggregated SNCA. SNO-SQSTM1/p62 blocks the fusion of autophagosomes and lysosomes, thus increasing accumulation of pathologically misfolded/oligomerized SNCA as well as its secretion in EVs. EV-mediated secretion of SNCA facilitates its cell-to-cell transmission, resulting in an increase in pathological aggregation of SNCA in recipient cells, and consequently contributing to the propagation of α-synucleinopathy.
